# Human mesenchymal stem cells improve rat islet functionality under cytokine stress with combined upregulation of heme oxygenase-1 and ferritin

**DOI:** 10.1186/s13287-019-1190-4

**Published:** 2019-03-12

**Authors:** Camille Laporte, Emily Tubbs, Justine Cristante, Anne-Sophie Gauchez, Sandra Pesenti, Frédéric Lamarche, Cécile Cottet-Rousselle, Catherine Garrel, Anaick Moisan, Jean-Marc Moulis, Eric Fontaine, Pierre-Yves Benhamou, Sandrine Lablanche

**Affiliations:** 1 Laboratory of Fundamental and Applied Bioenergetics (LBFA), INSERM U 1055 and SFR Environmental and Systems Biology (BEeSy), University Grenoble Alpes, Grenoble, BP 53, F-38041 Grenoble Cedex, France; 2 Grenoble University Hospital, Grenoble, France; 3 Biology Institute, Grenoble Alpes University Hospital, CS 10217, 38043 Grenoble Cedex 9, France; 4 Univ Lyon, CarMeN Laboratory, INSERM, INRA, INSA Lyon, Université Claude Bernard Lyon 1, 69600 Oullins, France; 5 Cell Therapy and Engineering Unit, EFS Auvergne-Rhône-Alpes, 464 Route de lancey - La Bâtie, 38330 Saint Ismier, France; 6 CEA-Grenoble, Bioscience and Biotechnology Institute (BIG), 38054 Grenoble, France

**Keywords:** Diabetes mellitus type 1, Islets of Langerhans transplantation, Mesenchymal stem cells, Co-culture, Cytokines, Heme oxygenase 1

## Abstract

**Background:**

Islets of Langerhans transplantation is a promising therapy for type 1 diabetes mellitus, but this technique is compromised by transplantation stresses including inflammation. In other tissues, co-transplantation with mesenchymal stem cells has been shown to reduce damage by improving anti-inflammatory and anti-oxidant defences. Therefore, we probed the protection afforded by bone marrow mesenchymal stem cells to islets under pro-inflammatory cytokine stress.

**Methods:**

In order to evaluate the cytoprotective potential of mesenchymal stem cells on rat islets, co-cultures were exposed to the interleukin-1, tumour necrosis factor α and interferon γ cocktail for 24 h. Islet viability and functionality tests were performed. Reactive oxygen species and malondialdehyde were measured. Expression of stress-inducible genes acting as anti-oxidants and detoxifiers, such as superoxide dismutases 1 and 2, NAD(P)H quinone oxidoreductase 1, heme oxygenase-1 and ferritin H, was compared to non-stressed cells, and the corresponding proteins were measured. Data were analysed by a two-way ANOVA followed by a Holm-Sidak post hoc analysis.

**Results:**

Exposure of rat islets to cytokines induces a reduction in islet viability and functionality concomitant with an oxidative status shift with an increase of cytosolic ROS production. Mesenchymal stem cells did not significantly increase rat islet viability under exposure to cytokines but protected islets from the loss of insulin secretion. A drastic reduction of the antioxidant factors heme oxygenase-1 and ferritin H protein levels was observed in islets exposed to the cytokine cocktail with a prevention of this effect by the presence of mesenchymal stem cells.

**Conclusions:**

Our data evidenced that MSCs are able to preserve islet insulin secretion through a modulation of the oxidative imbalance mediated by heme and iron via heme oxygenase-1 and ferritin in a context of cytokine exposure.

**Electronic supplementary material:**

The online version of this article (10.1186/s13287-019-1190-4) contains supplementary material, which is available to authorized users.

## Background

Islet transplantation is a cell therapy proposed to patients with brittle type 1 diabetes (T1D) experiencing severe hypoglycemia. Islet transplantation efficiency has been demonstrated to enhance glycemic control in T1D patients [1, 2], but several hurdles still need to be overcome. The amount of engrafted islets is a key point determining the graft outcome. Unfortunately, 50–70% of islet grafts are lost in the early post-transplant period due to various factors such as ischemia reperfusion, immunosuppressive therapy toxicity or instant blood-mediated inflammatory reaction mediated by pro-inflammatory cytokines [3].

Due to their poor oxidative defences [4, 5], islets are particularly sensitive to oxidative stress induced by inflammatory cytokines [6–8]. A recent study has shown that the overexpression of cytosolic superoxide dismutase (SOD1, a key antioxidant enzyme) in an insulin-secreting cell line enhanced cell viability after exposure to cytokines [9]. The Nrf2/ARE pathway, through the detoxifying enzymes NAD(P)H quinone oxidoreductase 1 (NQO1), a cytosolic two-electron reductase, and heme oxygenase-1 (HO-1), a ubiquitous enzyme identified as a stress-inducible antioxidant mediator, is implicated in the regulation of the oxidative status of islets [10, 11]. HO-1 is studied for its possible beneficial effect in transplantation [12]. Its overexpression by transfection or chemical induction leads to reduced islet sensitivity to oxidative stress induced by inflammatory cytokines [13–15].

Among strategies suggested to protect islets against inflammation and to enhance islet graft viability, islet co-transplantation with mesenchymal stem cells (MSCs) is a promising approach as MSCs have demonstrated a beneficial effect on islet survival and functionality in vitro [16, 17] and on the islet graft function in vivo [18–21]. MSCs partly prevent islet damage resulting from exposure to cytokines in vitro [22]. In vivo, MSCs reduce the inflammatory response in an intramuscular islet graft model [23]. Interestingly, MSCs are described to alleviate direct inhibition of antioxidant enzymes in different inflammatory diseases. Indeed, in a murine colitis model, it has been shown that MSC transplantation prevents the decrease in SOD1 activity induced by inflammation [24]. Similarly, MSCs upregulate HO-1 and NQO1 gene expression [24, 25] in various inflammatory disease models. In an islet transplantation model, it has been demonstrated that MSCs decrease reactive oxygen species (ROS) generation induced by hypoxia [26]. Although islet/MSC co-transplantation beneficial effects are well documented, the impact of MSCs on the antioxidant defences of islets exposed to inflammation has not been previously investigated.

Our aim was thus to assess the oxidative status of cytokine-exposed rat islets and to identify on which factors MSCs rely to preserve islet function and viability.

## Materials and methods

### Rat islet isolation procedure and animal approval

Rat islet isolation was performed in accordance with the European Council Directive 2010/63/UE and the recommendations of the French Ministry of Agriculture (2013-118) on the care and use of laboratory animals. Procedures were approved (n°9998_LBFA-U1055) by the local ethics committee and agreed by the French Ministry of Research. All protocols were carried out in the animal facility of the Department of Biology of the University (D3842110001). Rat islet isolation was performed as previously described [27]. Briefly, a cannula was inserted in the rat’s Wirsung duct to perfuse with collagenase type IX, 1 mg ml^− 1^ (Sigma-Aldrich, St. Louis, MO, USA). Perfused pancreas was removed and digested in a warm bath at 37 °C for 11 min. The digested pancreas was washed three times with a solution composed of Hank’s balanced salt solution (HBSS) (Pan Biotech, Aidenbach, Germany) supplemented with 10% of foetal bovine serum (FBS) (Sigma-Aldrich, St. Louis, MO, USA). Islets were purified by Histopaque (Sigma-Aldrich, St. Louis, MO, USA) density gradient centrifugation. After three additional washes by sedimentation to eliminate residual exocrine tissue, the islet pellet obtained is resuspended in 1 ml of RPMI 1640 medium (Thermo Fisher Scientific, Waltham, MA, USA) supplemented with 10% foetal bovine serum (Sigma-Aldrich, St. Louis, MO, USA), 2 mM L-glutamine, 100 U ml^− 1^ penicillin, 100 μg ml^− 1^ streptomycin and 1 mM sodium pyruvate (Pan Biotech, Aidenbach, Germany). One twentieth of this suspension is taken and seeded in a grid box containing 2 ml of medium and 200 μl of dithizone 0.4 mg ml^− 1^ in dimethyl sulfoxide (DMSO) solution. This solution permits to differentiate the endocrine tissue (i.e., the islets) from the exocrine tissue. Under microscopy, the islets are counted, measured and converted into islet equivalent (IEQ). One IEQ corresponds to the tissue volume of a perfectly spherical islet with a diameter of 150 μm. This procedure standardizes islet volume measurements and ensures a distribution of an equivalent islet volume between each condition. The rest of the islet suspension was seeded. Isolated islets were incubated at 37 °C in a humidified atmosphere (95% air, 5% CO_2_) for 12 h before co-culture with MSCs. Then IEQs were randomly distributed between each condition. The IEQ number used for each experiment varied in function of the amount necessary for the analysis.

### Human mesenchymal stem cell isolation and culture

MSCs were provided by the Cell Therapy and Engineering Unit of EFS Auvergne-Rhône-Alpes. Cells were isolated from the bone marrow aspirated from human healthy donors who gave their written informed consent. All procedures complied with the French public health code (Article L1241-1). MSC isolation was conducted as previously described [28]. After thawing, MSCs were seeded at 3500 cells/cm^2^. Cells were maintained in culture at 37 °C, 95% O_2_ and 5% CO_2_ in MEM alpha medium (Sigma-Aldrich, St. Louis, MO, USA) supplemented by 10% foetal bovine serum (Sigma-Aldrich, St. Louis, MO, USA), 2 mM Glutamax, 100 U/ml penicillin and 100 μg/ml streptomycin (Thermo Fisher Scientific, Waltham, MA, USA). The medium was changed every 3 to 4 days. Upon confluence, cells were dissociated with Trypsin-EDTA (0.25%) (Thermo Fisher Scientific, Waltham, MA, USA).

### Mesenchymal stem cell characterization

The Cell Therapy and Engineering Unit of EFS Auvergne-Rhône-Alpes provided pre-immunophenotyped MSCs with anti-CD45-FITC, anti-CD14-FITC, anti-CD105-PE, anti-CD90-APC and anti-CD73-PE-CY7 (Becton Dickinson Franklin Lakes, NJ, USA) which were analysed by flow cytometry according to the manufacturer’s instructions (data not shown). To confirm MSC functionality, cells were differentiated using induction media (Stempro differentiation kit, Thermo Fisher Scientific, Waltham, MA, USA) for adipocyte, chondrocyte and osteocyte formation and stained respectively with Oil Red O, Alcian blue and Alizarin Red S.

### Cell culture: HepG2 and A549

The cell lines, HepG2 derived from a human hepatocellular carcinoma and A549 from adenocarcinomic human alveolar basal epithelial, were selected for their human origin, for their proliferative velocity close to MSCs and for their non-pancreatic origin. The aim of these co-culture experiments was to determine if the protective effect observed is specific of MSCs and to rule out a protective effect due to unspecific cell-to-cell contact effect. The cells were provided by the Deutsche Sammlung von Mikroorganismen und Zellkulturen (DSMZ) culture collection. After thawing, HepG2 and A549 were cultivated as previously described [29, 30]. Briefly, HepG2 and A549 were seeded at a density of 2.7 × 10^4^ and 1.2 × 10^4^ cells/cm^2^ respectively. Cells were maintained in culture at 37 °C, 95% O_2_ and 5% CO_2_ in Dulbecco’s MEM (Thermo Fisher Scientific, Waltham, MA, USA) supplemented with 10% FBS (Sigma-Aldrich, St. Louis, MO, USA), 2 mM L-glutamine, 100 U/ml penicillin and 100 μg/ml streptomycin (Pan Biotech, Aidenbach, Germany). The medium was changed every 2 to 3 days. Upon confluence, cells were treated with Trypsin-EDTA (0.25%) (Thermo Fisher Scientific, Waltham, MA, USA).

### Co-culture and exposure to cytokines

MSCs, HepG2 and A549 were seeded 12 h before co-culture to allow cells to adhere. Cells were seeded at 100,000 cells for 200 islet equivalents (IEQs) to reach a ratio of 500 cells/IEQ in all cases. Rat islets were seeded in co-culture with MSCs, or with HepG2 or A549, and incubated in the complete RPMI medium for 24 h. For cytokine exposure, rat islets in co-culture with MSCs or not were incubated for 24 h in the complete RPMI medium supplemented with 600 UI ml^− 1^ of IL-1 β, 6000 UI ml^− 1^ of IFN-γ and 6000 UI ml^− 1^ of TNF-α (Merck Millipore, Burlington, MA, USA). The cytokine concentrations were determined through a dose-response analysis of islet viability exposed to different cytokine concentration. (Additional file 1). We selected the concentration inducing a significant but not drastic mortality of islets (20–40%).

### Assessment of islet viability

To discriminate viability of islet cells from MSCs, MSCs were stained with PKH67 before seeding islets, according to the manufacturer’s instructions (Sigma-Aldrich, St. Louis, MO, USA; *λ*exc\*λ*em = 490/504 nm). After cytokine exposure, islets co-cultured with MSCs were washed with PBS, dissociated with trypsin 1× and collected in PBS for a cytometric analysis of single-cell suspension. The two cell types were stained with 1.5 × 10^− 10^ mol mL^− 1^ of propidium iodide (PI) (Interchim, Montluçon, France), (*λ*ex/*λ*em = 545/630) allowing identification of the necrotic cells. Cells labelled with PI and PKH67 were considered as necrotic MSCs, unlabelled cells as viable islet cells and cells stained with PI alone as necrotic islet cells. The measurements were carried out using a FACS Calibur (BD Bioscience, San Jose, CA, USA) instrument.

### Glucose-stimulated insulin secretion test

To assess the insulin secretion capacity of islets, we performed a glucose-stimulated insulin secretion (GSIS) test adapted from a previously described protocol [31]. For each condition tested, 200 rat IEQs were incubated at 37 °C, 95% O_2_ and 5% CO_2_ for 1 h in 2 ml KREBS buffer 1% BSA and 2.8 mM glucose. Thereafter, islets were incubated for 1 h with 2.8 mM glucose solution and for 1 h with 16.7 mM glucose solution. After each incubation step, supernatants were collected. Insulin concentration in each collected medium was measured using a commercial radioimmunoassay kit for rat insulin (Merck Millipore Corporation, Germany). Insulin measurements were performed by the Grenoble Biology and Pathology Institute.

### Measurement of cytosolic and mitochondrial reactive species in islet cells

One hundred IEQs were dissociated with Trypsin-EDTA (0.25%) and incubated for 30 min with 25 μM of Carboxy-H2DCFDA (*λ*exc\*λ*em = 504/529 nm) (Interchim, Montluçon, France) or 5 μM of MitoSOX red (*λ*exc\*λ*em = 510/580 nm) (Invitrogen, Carlsbad, CA, USA). Carboxy-H2DCFDA is a non-specific indicator of the cytosolic reactive oxygen species (ROS) and reactive nitrogen species (RNS) such as hydrogen peroxide (H_2_O_2_) and peroxynitrite anion (ONOO^−^). MitoSOX Red staining is an indicator of the production of superoxide (O_2_^−^) generated inside the mitochondria. The ROS content of each cellular compartment was expressed as average signal intensity measured by flow cytometry.

### Lipid peroxidation measurement

Lipid peroxidation was quantified by measuring malondialdehyde (MDA). Briefly, 400 rat IEQs were disintegrated by 5 freezing/thawing cycles in 250 μl of Tris hypotonic buffer. TBA reactive substances, including peroxidized lipids, were assayed using the 2-thiobarbituric acid method as described previously [32].

### RT-qPCR

Total RNA from 200 rat IEQs was purified using the TriReagent kit (Applied Biosystems, Foster City, CA, USA). Relative contents of target mRNA levels were determined by reverse transcription followed by real-time PCR using a Rotor-GeneTM 6000 (Corbett Research, Saffron Walden, UK) with the primers reported in Additional file 2. Each assay was performed in duplicate, and values were normalized using hypoxanthine-guanine phosphoribosyltransferase (HPRT).

### Western blotting

Four hundred rat IEQs were lysed in RIPA buffer (Sigma-Aldrich, St. Louis, MO, USA) supplemented with protease inhibitor cocktails (Roche, Basel, Switzerland). Proteins were separated by SDS-10% PAGE (Biorad), transferred to nitrocellulose membranes and incubated overnight with the following primary antibodies: anti-superoxide dismutase 1 (SOD1) (1:2000), anti-NAD(P)H dehydrogenase (quinone 1) (NQO1) (1:1000), anti-heme oxygenase 1 (HO-1) (1:1000) and anti-ferritin H (FTH) (1:1000) (Abcam, Cambridge, UK). The signals were detected with horseradish peroxidase-conjugated secondary antibodies: goat anti-rabbit IgG (1:10,000) and goat anti-mouse IgG (Jackson ImmunoResearch, West Grove, PA, USA), and revealed with an enhanced chemiluminescence system (Thermo Fisher). Due to our experimental conditions mimicking inflammation, we were unable to find a stable protein as a loading control. To address this issue, blot signals were normalized with Ponceau S staining of each lane.

### SOD activity assessment

For each condition, 800 rat IEQs were harvested and disintegrated by 5 freezing/thawing cycles in 250 μl of Tris hypotonic buffer. Total SOD, SOD1 and SOD2 activities were measured as described previously [32] using the pyrogallol assay.

### Statistical analysis

To analyse MSCs’ impact on cytokine-exposed islets, data from exposure to cytokine conditions were normalized on control values. All the data were expressed as median ± interquartile and represented in box plots. When data were normally distributed, a two-way ANOVA was performed. If significant, the omnibus test of variance was followed by a Holm-Sidak post hoc analysis. For non-parametric data, a log or square root transformation was performed before the two-way ANOVA. The threshold for significance was set at *p* < 0.05.

## Results

### MSCs exhibit conformity to phenotype and functionality

MSCs positive for CD44, CD73, CD90 and CD105 were able to differentiate into adipocytes (stained by Oil Red O), chondrocytes (stained by Alcian blue) and osteocytes (stained by alizarin Red S) demonstrating their proper functionality (Additional file 3).

### MSCs co-cultured with islets protect insulin secretion but not islet viability under exposure to cytokines

**Fig. 1:**
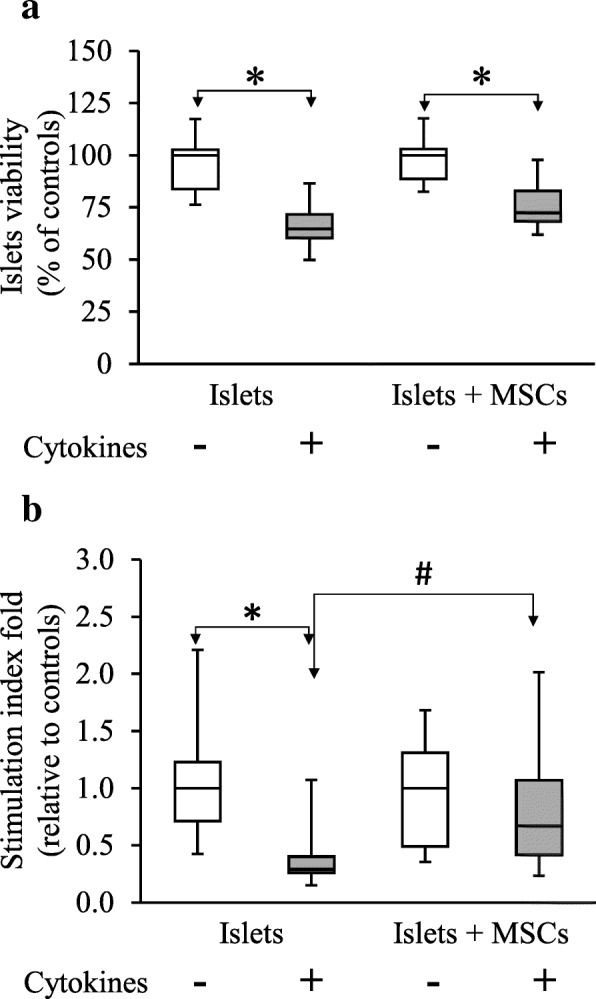
Mesenchymal stem cells effect on rat islet viability and insulin secretion after exposure to cytokines. **a** Islet viability was assessed by flow cytometry after PKH67/Propidium iodide double staining. Data are representative of six independent experiments and are represented as median ± interquartile (ANOVA; *p < 0.05 vs. respective controls and #*p* < 0.05 vs. cytokine-exposed islets alone). **b** Stimulation index was determined based on the insulin secretion response to glucose stimulation. Data are representative of 10 independent experiments and are represented as median ± interquartile

After exposure to cytokines, we observed a significant decrease in islet viability with an islet viability of 64.8% [60.4%–71.7%] in islets exposed to cytokines vs. 100% [83.8–102.6%] in control islets (*p* < 0.001; *n* = 6). Moreover, cytokine exposure induced a significant reduction of glucose-stimulated insulin secretion with an islet stimulation index fold of 0.29 [0.26–0.40] in islets exposed to cytokines vs. 1 [0.71–1.23] in control islets (Fig. 1). (*p* < 0.001; *n* = 10) (Fig. 1).

MSCs co-cultured with islets did not statistically impact viability of islets exposed to cytokines (72.5% [68.4–82.9%]) compared to islets alone exposed to cytokines (64.8% [60.4–71.7%]) (*p* = 0.21; *n* = 6) (Fig. 1a). Nevertheless, co-culture of islets with MSCs enhanced statistically the stimulation index from 0.29 [0.26–0.40] in islets alone exposed to cytokines to 0.67 [0.41–1.07] in islets co-cultured with MSCs and exposed to cytokines (*p* = 0.004; *n* = 10) (Fig. 1b).

The positive effect observed on islet insulin secretion is specific of MSC co-culture: no enhancement of insulin secretion was observed when islets were co-cultured with HepG2 and A549 cell lines seeded at the same density (see Additional file 4).

### MSCs partially restore antioxidant and anti-inflammatory protein levels in islets under cytokine exposure

**Fig. 2:**
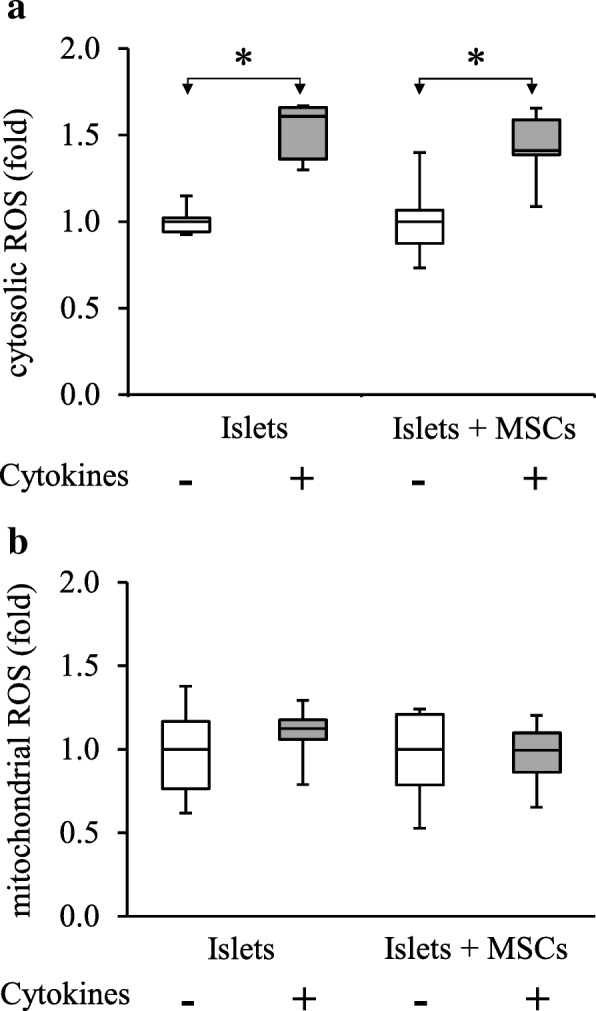
Oxidative status evaluation of islets in different culture conditions. **a** Cytosolic and **b** mitochondrial ROS assessment were performed by flow cytometry using respectively Carbo-H2DCFDA and MitoSOX probes. Data are representative of five independent experiments and are represented as median ± interquartile (ANOVA; *p < 0.05 vs. respective controls)

We investigated whether the implemented inflammatory conditions were associated with a change in islet oxidative status. While mitochondrial superoxide production measured by MitoSOX fluorescence was not affected by exposure to cytokines (Fig. 2b), we observed a significant increase of cytosolic oxidative species from 1 [0.94–1.02] in islets not exposed to cytokines to 1.61 [1.36–1.65] in islets exposed to cytokines (*p* < 0.002; *n* = 5, Fig. 2a).

MDA measurements showed that the increase of cytosolic oxidative species did not correlate with the increase of thiobarbituric acid reactive species (Additional file 5), implying that lipid peroxidation, a marker of advanced cell damage, is not significantly induced by the cytokine stress.

MSCs co-cultured with islets did not prevent islet cytosolic ROS and RNS production induced by cytokine exposure (1.4 [1.4–1.6]) (*p* = 0.459; *n* = 5) (Fig. 2a, b). MDA analysis confirmed the absence of advanced detrimental oxidative effect of exposure to cytokines also in MSC co-cultures (Additional file 5).

**Fig. 3:**
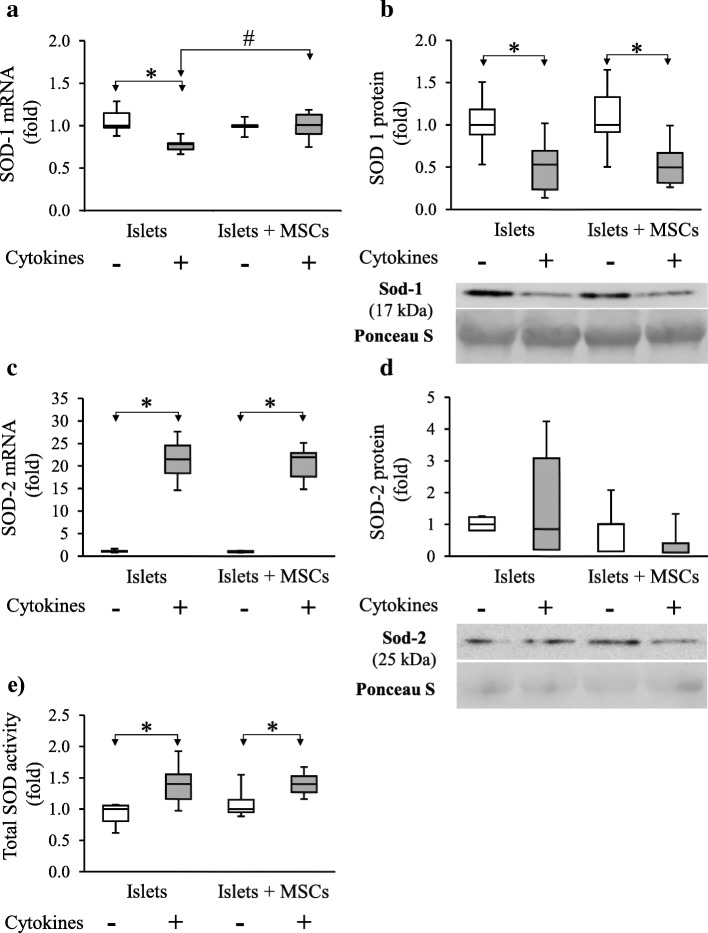
MSCs’ effect on cytokine-exposed rat islet’s superoxide dismutases 1 and 2. **a**, **c** Transcripts were measured by RT-PCR and values were normalized on HPRT. **b**, **d** Quantitative analysis of proteins normalized with Ponceau S staining of the whole lane. **e** SOD activity was determined using the pyrogallol assay. Data are representative of six independent experiments for RT-PCR analysis and eight for western blot analysis and SOD activity. Data are represented as median ± interquartile (ANOVA; *p < 0.05 vs. respective controls and #*p* < 0.05 vs. cytokine-exposed islets alone)

To confirm the pro-oxidative shift in islets induced by cytokines, we studied the islet antioxidant response following cytokine exposure. Regarding direct antioxidant defences, cytokine exposure induced a significant decrease of SOD1 mRNA from 1 [0.98–1.15] in control islets to 0.78 [0.72–0.80] (*p* < 0.001; *n* = 6) in cytokine-exposed islets. A corresponding significant SOD-1 protein level decrease is observed from 1 [0.89–1.18] in control islets to 0.53 [0.24–0.69] in cytokine-exposed islets (*p* = 0.006; *n* = 8) (Fig. 3a, b). Cytokine exposure induced a strong and statistically significant SOD2 mRNA overexpression from 1 [0.96–1.16] in control islets to 21.49 [18.42–24.54] in cytokine-exposed islets (*p* < 0.001; *n* = 6) (Fig. 3c). However, no change in SOD2 protein levels was observed in cytokine-exposed islets as compared to control islets (Fig. 3d). The total SOD activity statistically increased from 1 [0.81–1.06] in control to 1.40 [1.16–1.56] in cytokine-exposed islets (*p* = 0.003; *n* = 8) (Fig. 3e).

Regarding the MSCs’ impact on islet antioxidant response under cytokine exposure, the presence of MSCs restored islet SOD 1 gene expression close to the SOD1 level of control islets (1.01 [0.90–1.13] in islets co-cultured with MSCs and exposed to cytokines vs. 1 [0.99–1.01] in control islets (*p* = 0.94; *n* = 6)) (Fig. 3a). No effect of MSCs on SOD2 gene expression was observed (Fig. 3c). Under cytokine exposure, MSC co-culture did not impact islet SOD1 and SOD2 protein levels nor SOD activity as compared to islets alone exposed to cytokines (Fig. 3b, d, e).

**Fig. 4:**
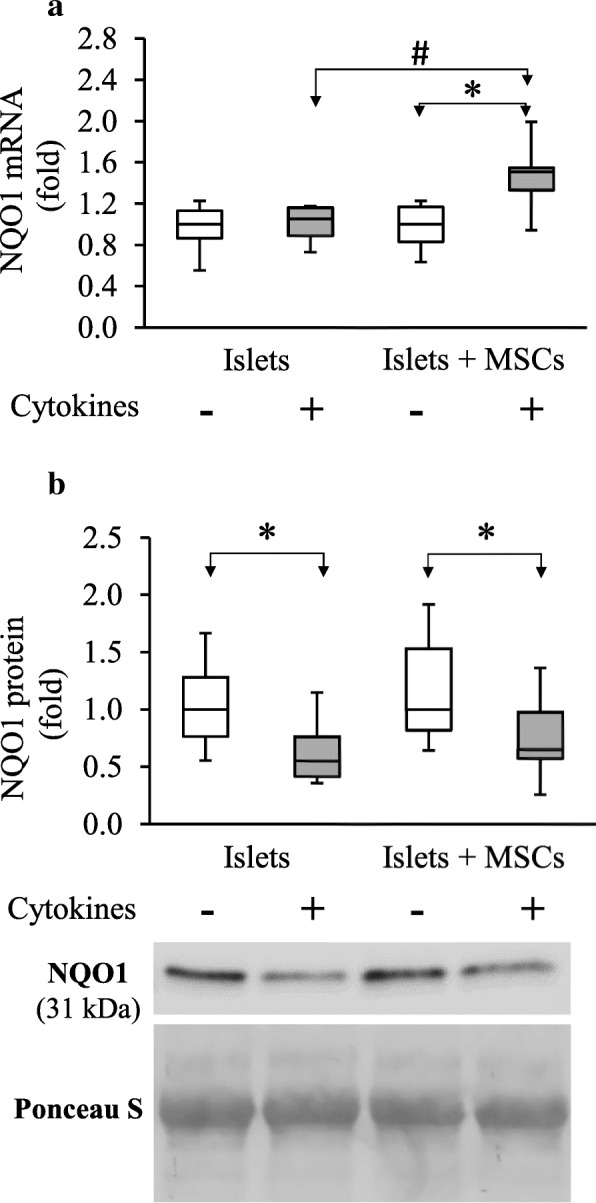
MSCs’ effect on cytokine-exposed rat islet’s NAD(P)H dehydrogenase (quinone 1) expression. **a** Transcripts were measured by RT-PCR and values were normalized on HPRT. **b** Blot signals were normalized with Ponceau S staining of the whole. Data are representative of six independent experiments for RT-PCR analysis and eight for western blot analysis and are represented as median ± interquartile (ANOVA; *p < 0.05 vs. respective controls and #*p* < 0.05 vs. stressed islets alone)

Regarding NQO1, in cytokine-exposed islets, no change in gene expression was observed as compared to control islets, whereas NQO1 protein levels statistically decreased after cytokine exposure (0.55 [0.41–0.76]) as compared to control islets (1.00 [0.77–1.28]) (*p* = 0.024; *n* = 8) (Fig. 4a, b). NQO1 gene expression in islets co-cultured with MSCs and exposed to cytokines was statistically increased (1.51 [1.33–1.55]) as compared to islets alone exposed to cytokines (1.05 [0.89–1.16]; *p* = 0.006; *n* = 6) (Fig. 4a). No significant difference on the NQO1 protein level in islets co-cultured with MSCs exposed to cytokines was observed as compared to islets alone exposed to cytokines (*p* = 0.469; *n* = 8) (Fig. 4b).

**Fig. 5:**
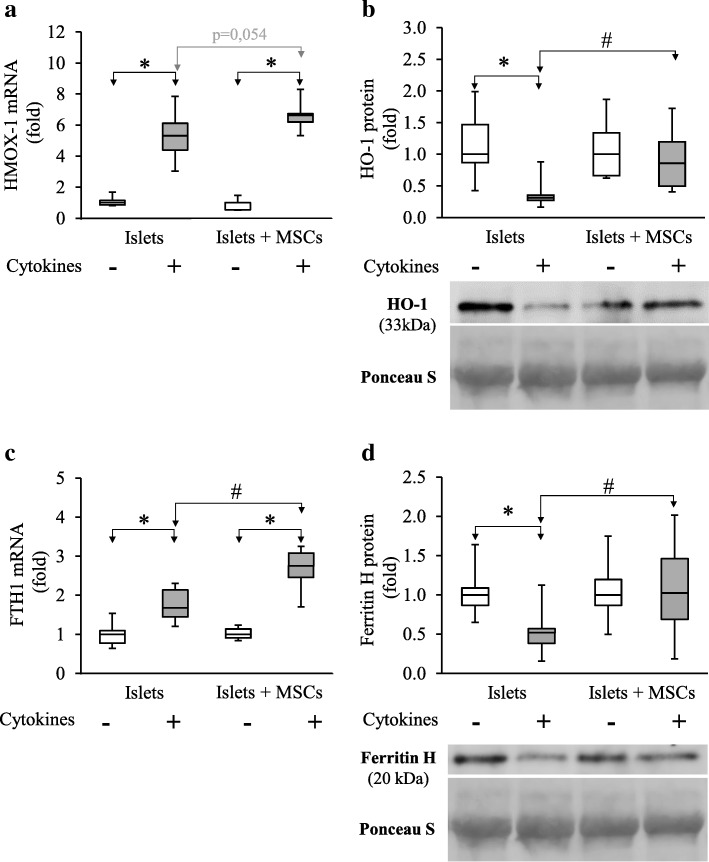
MSCs stimulate heme oxygenase 1 and the downstream ferritin heavy-chain subunit in cytokinic stress conditions. **a**, **c** Transcripts were measured by RT-PCR and values were normalized on HPRT. **b**, **d** Blot signals were normalized with Ponceau S staining of the whole lane. Data are representative of six independent experiments for RT-PCR analysis and eight for western blot analysis and are represented as median ± interquartile (ANOVA; *p < 0.05 vs. respective controls and #*p* < 0.05 vs. stressed islets alone)

The HMOX1 mRNA level was significantly and strongly increased in cytokine-exposed islets (5.70 [4.63–6.15]) as compared to control islets (1.00 [0.94–1.22]; *p* < 0.001; *n =* 6) (Fig. 5a). However, a significant decrease in the HO-1 protein level was observed in cytokine-exposed islets (0.31 [0.27–0.35]) as compared to control islets (1.00 [0.87–1.47]; *p* < 0.001; *n =* 8) (Fig. 5b). In the same way, the FTH1 mRNA level increased statistically in islets alone exposed to cytokines (1.68 [1.44–2.14]) compared to control islets (1.00 [0.78–1.10]; *p* = 0.004; *n =* 6) while a significant decrease in ferritin H protein levels was observed in islets alone exposed to cytokines (0.52 [0.38–0.57]) vs. control islets (1.00 [0.87–1.09]; *p* = 0.01; *n =* 8) (Fig. 5c, d).

In co-culture with MSCs, HMOX1 mRNA expression tended to increase from 5.70 [4.63–6.15] in islets alone exposed to cytokines to 6.64 [6.21–6.74] in islets co-cultured with MSCs exposed to cytokines (*p* = 0.054; *n =* 6). HO-1 protein levels were significantly increased in islets co-cultured with MSCs exposed to cytokines (0.86 [0.50–1.20]) as compared to islets alone exposed to cytokines (0.31 [0.27–0.35]; *p* = 0.012; *n =* 8) (Fig. 5a, b). Similarly, FTH1 mRNA expression was statistically increased in islets co-cultured with MSCs exposed to cytokines (2.76 [2.46–3.08]) as compared to islets alone exposed to cytokines (1.68 [1.44–2.14]; *p* < 0.001; *n =* 6). The ferritin H protein level was statistically increased in islets co-cultured with MSCs exposed to cytokines (1.02 [0.69–1.46]) as compared to islets alone exposed to cytokines (0.52 [0.38–0.57], *p* = 0.019, *n =* 8) (Fig. 5c, d).

## Discussion

In this study, we showed that the loss of insulin secretion induced by exposure to cytokines was partially prevented by MSC co-culture. This effect is specific to MSCs as evidenced by the results of co-culture with two unrelated cell lines. MSCs helped islets in maintaining the HO-1 and ferritin H protein levels under exposure to cytokines which otherwise fell under this stress. These results suggest that the HO-1/ferritin H pathway participates in the protective effect of MSCs on islet functionality.

We demonstrated that under exposure to cytokines, co-cultured islets exhibited better insulin secretion capability than control islets, while no effect was observed on islet viability. If MSCs’ positive effect on islet functionality in vitro is widely described [17], data on viability of islets with MSCs are discordant and need to be further explored. Some studies evidenced a positive impact of MSCs on in vitro islet viability [22] whereas others reported no differences on in vitro islet viability when co-cultured with MSCs [33] probably depending on cell culture conditions (time of culture, direct cell contact vs. indirect co-culture system, stress induction) as well as source of MSCs.

It was demonstrated that insulin secretion is widely impacted by an overproduction of ROS in an inflammatory context [34]. In our study, cytokine exposure exerts a slight cytosolic but not mitochondrial ROS overproduction. Regarding co-culture with MSCs, in our 24-h cytokine exposure model, MSCs have no effect on islet cytosolic ROS elevation. While in our model, cytosolic ROS overproduction seems to not induce advanced oxidative damages as illustrated by lipid peroxidation measurements, cytokine exposure induces an oxidative status shift that is confirmed by an antioxidant enzyme response.

Regarding SODs, we observed two different response patterns. SOD1 mRNA and protein are slightly downregulated by cytokine exposure as previously described in other stress models [35, 36]. On the other hand, SOD2 seems to be more sensitive and islet cytokine exposure results in a rapid increase of its transcriptional and translational level as previously described [35, 36]. In our model, the presence of MSCs only restores the transcriptional level of SOD1 with no effect on SOD2 gene, protein expression nor activity.

Few studies have been conducted to evaluate the role of NQO1 in the mechanisms involved in the islet protection against oxidative stress [37]. It was reported that the protective effect of curcumin and Nrf2 activation induced by dh404 is accompanied by an increase in NQO1 expression at the mRNA and protein level [10, 38]. Interestingly, in our model, NQO1 gene expression seems to be naturally non-sensitive to cytokine exposure. On the other hand, MSCs modestly stimulate NQO1 mRNA expression. This observation is consistent with the literature that showed, in a pulmonary fibrosis model, an upregulation of NQO1 mRNA in the presence of MSCs [14]. However, regarding protein regulation in our model, cytokine exposure induced a slight NQO1 decrease not prevented by the MSC co-culture.

The difference between transcriptional response and translational response of the different enzymes exposed to cytokines and in the presence of MSCs has to be noted. These observations could suggest the establishment of post-transcriptional regulations that are not prevented by the presence of MSCs or a longer kinetic of regulation longer preventing the observation of the MSC effect at a protein level.

In our study, we demonstrated that MSCs induced preservation of HO-1 and ferritin H protein levels in islets exposed to cytokines. Our data are consistent with the literature evidencing an impact of MSCs on HO-1 upregulation in different cells and stress conditions. Specifically, MSCs are described to damper inflammation in a rat model of acute liver failure by inducing HO-1 upregulation [25]. In the same way, a study showed upregulation of HO-1 by MSCs in a pulmonary fibrosis model [39]. In islets, MSCs improved islet resistance against hypoxia/reoxygenation through increase of HO-1 expression [40].

HO-1 upregulation has been previously described to confer anti-apoptotic and anti-inflammatory properties to various cell lines and tissues including islets. In islets, HO-1 upregulation or overproduction has been described to enhance insulin release of islets in vitro [41] and to enhance islet graft function in vivo [42]. HO-1 degrades heme, a strong oxidative moiety when released in cells, into ferrous iron, biliverdin (BV) and carbon monoxide (CO). These last two heme catabolites have been described in previous publications [43] for their own antioxidant, anti-inflammatory and anti-apoptotic properties. BV reductase converts BV in bilirubin (BR) that showed anti-inflammatory properties in an islet isolation and transplantation procedure [44, 45]. CO is a cell signalling activator of anti-inflammatory pathways by modulating guanylate cyclase and activating the mitogen-activated protein kinase (MAPK) pathways [46]. Consequently, considering the negative impact of inflammation on islet insulin secretion [47], HO-1 could improve inflammation status of the islets and subsequent insulin secretion capacity by activating the anti-inflammatory response. Moreover, the HO-1/CO pathway was identified as an insulin secretion stimulator by the activating the cGMP pathway [48], and CO was described to act as a messenger that potentiates insulin secretion by propagating Ca^2+^ signals between β cells [49]. Our results show that MSCs contribute to maintain the insulin secretion capacity observed in islets exposed to cytokines through enhanced HMOX1 expression and preservation of HO-1 levels.

Mechanisms by which MSCs restore HO-1 protein levels need to be clarified. It was demonstrated that HO-1 protein levels were regulated at the post-transcriptional level by nitric oxide (NO) that stabilizes HO-1 mRNA by preventing its deadenylation [50] thus enhancing the mRNA half-life and, consequently, translation. In preliminary experiments, we observed that exposure to cytokines upregulated inducible nitric oxide synthase (iNOS) mRNA in islets (see Additional file 6), but no differences in iNOS mRNA levels were observed in the presence of MSCs. Thus, the restoration of the HO-1 protein level in the presence of MSCs is unlikely to be mediated by NO. The pathways by which ferritin H is regulated are extensively described [51]. FTH1 is transcriptionally controlled by Nrf2 which may explain the concomitant increase of FTH1 mRNA and HO-1 expression with NQO-1 expression in our study. Further experiments analysing the Nrf2 pathway activation could be of interest to clarify its involvement in the MSC protective effects. The translation of ferritin H is inhibited by the iron regulatory proteins (IRP) which bind to the iron regulatory elements (IRE) located in the 5′ untranslated region of the mRNA of the ferritin subunits [46]. The combined observations of HMOX-1 overexpression and the preservation of the protein level in the presence of MSCs under the cytokine stress (Fig. 5a, b) may signal the need for sustained activity, hence significant production of the substrates. Among them, liberated ferrous iron must be properly handled by cells to avoid increased oxidative stress. This is one of the main functions of ferritin in agreement with the preservation of its level in the presence of MSCs (Fig. 5c, d). The decreased or preserved IRP activity associated with the release of iron by heme oxygenase may thus contribute to the maintenance of the ferritin levels in an exquisitely sensitive phenomenon occurring with minor variations of the iron concentrations [52]. This provides another regulatory mechanism at the translational level which can complete the transcriptional one afforded by Nrf2 to keep ferritin at a level allowing it to protect islets under the cytokine challenge.

Here, we analysed the impact of MSC on antioxidant defences in an in vitro model. Effects of MSC on islet, in vivo, are multifactorial and have been previously described with paracrine effects [22], anti-inflammatory effects [23], pro-angiogenic effects [53] and immunomodulatory effects [54]. The anti-oxidant effects participate, among the others, to the positive effects of MSCs. Future experiments should be performed, in vivo, to analyse in a model of islet transplantation the effect of MSCs on oxidative status of islets.

## Conclusions

To conclude, our data evidenced that MSCs are able to enhance islet insulin secretion in vitro under cytokine exposure and to preserve HO-1 and ferritin H protein levels. This work opens new perspectives in the multiple mechanisms underlying MSC protection. It highlights the impact of MSCs on islet oxidative imbalance. Further in vivo studies will be necessary to analyse the impact of antioxidant defence modulation by MSCs on islet physiology.

## Additional files

Additional file 1: Dose-response analysis of islet viability exposed to different cytokine concentration. Islet viability was assessed by flow cytometry after propidium iodide staining. Data are representative of three independent experiments and are represented as average ± SEM (ANOVA; *: *p* < 0.05 vs. controls). (PDF 245 kb)

Additional file 2: Forward and reverse primers used for qRT-PCR. (PDF 229 kb)

Additional file 3: Mesenchymal stem functionality (a) Undifferentiated MSCs (b) MSC-derived osteocytes (Alizarin red staining). (b) MSC-derived adipocytes (Oil Red O staining). (c) MSC-derived chondrocytes (Alcian blue staining). Scale bar = 50 μm. (PDF 241 kb)

Additional file 4: HepG2 and A549 effect on rat insulin-secretory function after exposure to cytokines. Stimulation index was determined based on the insulin-secretory response to glucose stimulation. Data are representative of seven independent experiments (*: *p* < 0.05 vs. respective controls). (PDF 171 kb)

Additional file 5: Lipid peroxidation evaluation of islets alone or islets co-cultured with MSCs. Lipid peroxidation was studied by MDA measurements. Data are representative of eight independent experiments. (PDF 253 kb)

Additional file 6: Impact of exposure to cytokines and MSCs’ influence on inducible nitrite oxide synthase mRNA form. Transcripts were measured by RT-PCR and values were normalized on HPRT. iNOS mRNA is widely upregulated by cytokinic stress in islets alone and islets in co-culture with MSCs. Data are representative of six independent experiments (*: *p* < 0.05 vs. respective controls). (PDF 256 kb)

## Abbreviations

ARE: Antioxidant response element
BR: Bilirubin
BV: Biliverdin
cGMP: Cyclic guanosine monophosphate
CO: Carbon monoxide
DMSO: Dimethyl sulfoxide
FBS: Foetal bovine serum
Ferritin H: Ferritin heavy chain (protein form)
FTH1: Ferritin heavy chain 1 (gene form)
H_2_O_2_: Hydrogen peroxide
HBSS: Hank’s balanced salt solution
HMOX1: Heme oxigenase 1 (gene)
MSC: Mesenchymal stem cells
HO^−^: Hydroxyl radical
HO-1: Heme oxygenase 1 (protein)
IEQ: Islet equivalent
iNOS: Inducible nitric oxide synthase
IRE: Iron regulatory elements
IRP: Iron regulatory proteins
MAPK: Mitogen-activated protein kinases
MDA: Malondialdehyde
MSC: Mesenchymal stem cell
NO: Nitric oxide
NQO1: NAD(P)H quinone dehydrogenase 1
Nrf2: Nuclear factor E2-related factor 2
O_2_^−^: Superoxide
ONOO^−^: Peroxynitrite anion
PI: Propidium iodide
RNS: Reactive nitrogen species
ROS: Reactive oxygen species
SOD: Superoxide dismutase
