# Supplementary figures and images for: Human mesenchymal stem cells improve rat islet functionality under cytokine stress with combined upregulation of heme oxygenase-1 and ferritin
Source: Stem Cell Res Ther. 2019 Mar 12;10:85. doi: 10.1186/s13287-019-1190-4 (PMC6416979; doi:10.1186/s13287-019-1190-4)

# Additional file 1.

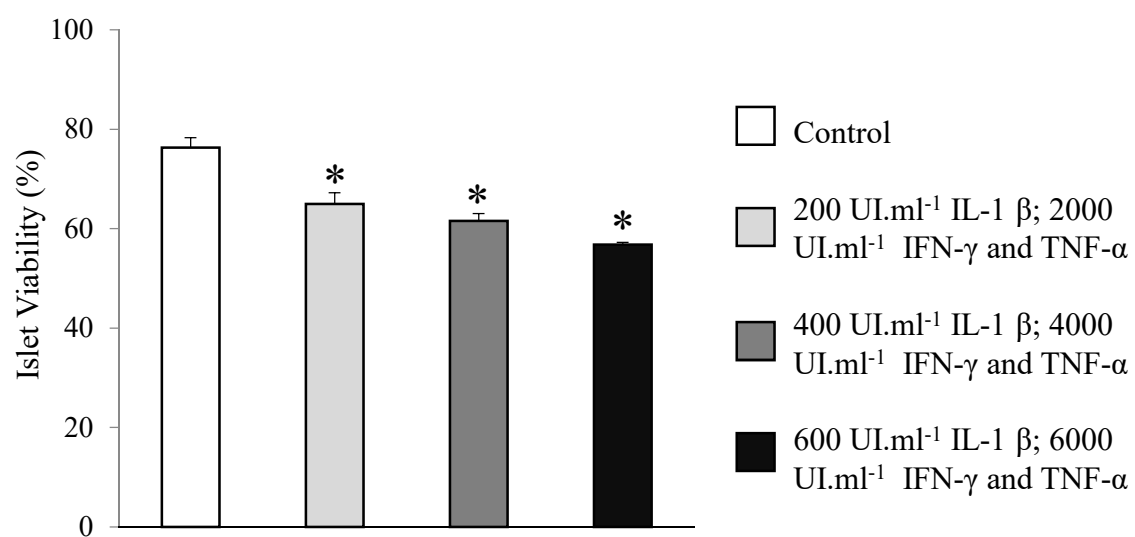

Supplement: Supplementary file 1 — Dose-response analysis of islet viability exposed to different cytokine concentration. Islet viability was assessed by flow cytometry after propidium iodide staining. Data are representative of three independent experiments and are represented as average ± SEM (ANOVA; *: p < 0.05 vs. controls). (PDF 245 kb) [file 13287_2019_1190_MOESM1_ESM.pdf]

### Additional file 3.

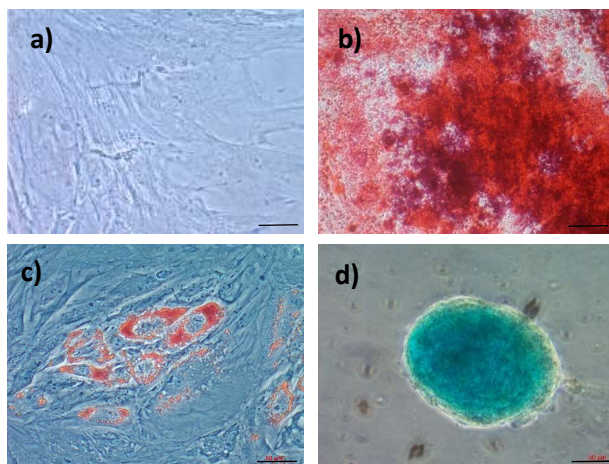

Supplement: Supplementary file 3 — Mesenchymal stem functionality (a) Undifferentiated MSCs (b) MSC-derived osteocytes (Alizarin red staining). (b) MSC-derived adipocytes (Oil Red O staining). (c) MSC-derived chondrocytes (Alcian blue staining). Scale bar = 50 μm. (PDF 241 kb) [file 13287_2019_1190_MOESM3_ESM.pdf]

**Additional file 4.**

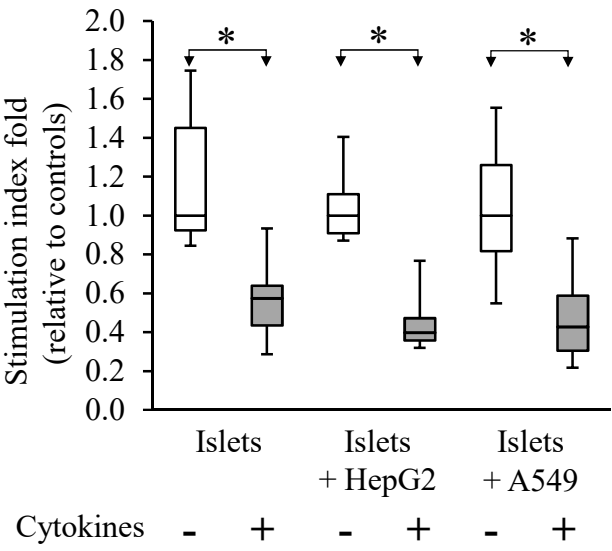

Supplement: Supplementary file 4 — HepG2 and A549 effect on rat insulin-secretory function after exposure to cytokines. Stimulation index was determined based on the insulin-secretory response to glucose stimulation. Data are representative of seven independent experiments (*: p < 0.05 vs. respective controls). (PDF 171 kb) [file 13287_2019_1190_MOESM4_ESM.pdf]

**Additional file 5.**

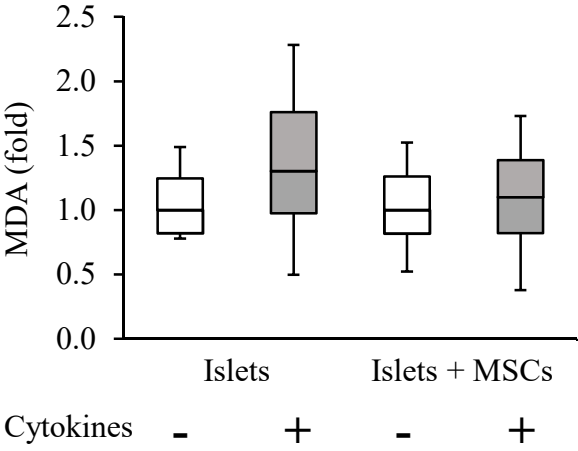

Supplement: Supplementary file 5 — Lipid peroxidation evaluation of islets alone or islets co-cultured with MSCs. Lipid peroxidation was studied by MDA measurements. Data are representative of eight independent experiments. (PDF 253 kb) [file 13287_2019_1190_MOESM5_ESM.pdf]

## Additional file 6.

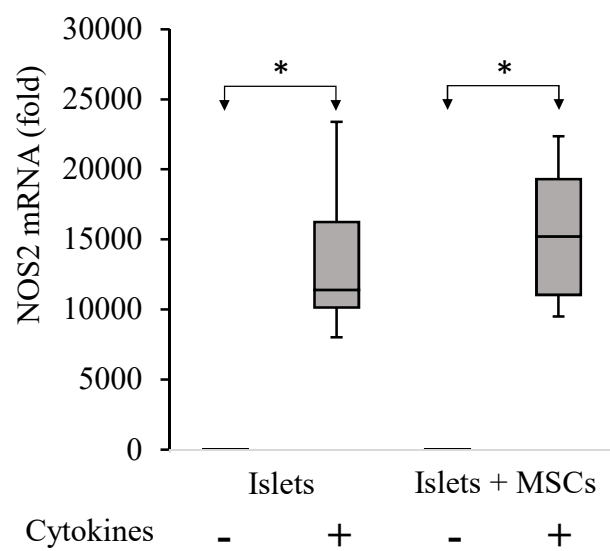

Supplement: Supplementary file 6 — Impact of exposure to cytokines and MSCs’ influence on inducible nitrite oxide synthase mRNA form. Transcripts were measured by RT-PCR and values were normalized on HPRT. iNOS mRNA is widely upregulated by cytokinic stress in islets alone and islets in co-culture with MSCs. Data are representative of six independent experiments (*: p < 0.05 vs. respective controls). (PDF 256 kb) [file 13287_2019_1190_MOESM6_ESM.pdf]
