# Supplementary material for: Human mesenchymal stem cells improve rat islet functionality under cytokine stress with combined upregulation of heme oxygenase-1 and ferritin
Source: Stem Cell Res Ther. 2019 Mar 12;10:85. doi: 10.1186/s13287-019-1190-4 (PMC6416979; doi:10.1186/s13287-019-1190-4)
Supplement: Supplementary file 2 — Forward and reverse primers used for qRT-PCR. (PDF 229 kb) [file 13287_2019_1190_MOESM2_ESM.pdf]

Additional file 2.

Table 1. Forward and Reverse primers used for qRT-PCR

Names	Forward primer	Reverse primer
**SOD 1**	TGA AGA GAG GCA TGT TGG AG	CCA CCT TTG CCC AAG TCA TC
**SOD 2**	TCA TGC AGC TGC ACC ACA GC	CCA TTG AAC TTC AGT GCA GG
**mHMOX1**	CAA GCA CAG GGT GAC AGA AG	TAC ATG GCA TAA ATT CCC ACT
**mNQO1**	TGC AGA CCT GGT GAT ATT TC	GGT TCT AAG ACC TGG AAG CC
**FTH1**	CAT CAA CCG CCA GAT CAA CC	TGC ACA CTC CAT TGC ATT C
**HPRT**	TTG CTG ACC TGC TGG ATT AC	AGT TGA GAG ATC ATC TCC AC
